# Synthesis of Quercetin Loaded Nanoparticles Based on Alginate for Pb(II) Adsorption in Aqueous Solution

**DOI:** 10.1186/s11671-015-1117-7

**Published:** 2015-10-17

**Authors:** Yun Qi, Meng Jiang, Yuan-Lu Cui, Lin Zhao, Xia Zhou

**Affiliations:** Faculty of Environmental Science and Engineering, Tianjin University, No. 92, Weijin Rd., Nankai District, Tianjin, 300072 China; School of Biological Sciences (A12), University of Sydney, Sydney, NSW 2006 Australia; Tianjin State Key Laboratory of Modern Chinese Medicine, Tianjin University of Traditional Chinese Medicine, Tianjin, 300193 China

**Keywords:** Pb(II), Alginate, Quercetin, Nanoparticles, Adsorption

## Abstract

Pb(II) is a representative heavy metal in industrial wastewater, which may frequently cause serious hazard to living organisms. In this study, comparative studies between alginate nanoparticles (AN) and quercetin-decorated alginate nanoparticles (Q-AN) were investigated for Pb(II) ion adsorption. Characterization of AN and Q-AN were analysed by transmission electron microscopy (TEM), Fourier transform infrared spectrometry (FT-IR), X-ray diffractometer (XRD), and thermogravimetric analysis (TG-DTG-DSC). The main operating conditions such as pH, initial concentration of Pb(II), and co-existing metal ions were also investigated using a batch experiment. AN and Q-AN, with a diameter of 95.06 and 58.23 nm, were constituted by many small primary nanoparticles. It revealed that when initial concentration of Pb(II) is between 250 and 1250 mg L^−1^, the adsorption rate and equilibrium adsorption were increased with the increase of pH from 2 to 7. The maximum adsorption capacities of 147.02 and 140.37 mg L^−1^ were achieved by AN and Q-AN, respectively, with 0.2 g adsorbents in 1000 mg L^−1^ Pb(II) at pH 7. The adsorption rate of Pb(II) was little influenced by the co-existing metal ions, such as Mn(II), Co(II), and Cd(II). Desorption experiments showed that Q-AN possessed a higher desorption rate than AN, which were 90.07 and 83.26 %, respectively. AN and Q-AN would probably be applied as adsorbents to remove Pb(II) and then recover it from wastewater for the advantages of simple preparation, high adsorption capacity, and recyclability.

## Background

The unique nature of lead (Pb), high malleability, low melting point, and strong resistance to corrosion, provides its widespread application in various industries [1]. The major sources of anthropogenic Pb are the wastewaters from battery manufacturing, electroplating industry, mining activity, and combustion of automobile petrol [2, 3]. Pb(II) has access to human body through inhalation and ingestion in different ways such as contaminated air and water, soil, and food. Accumulation of lead produces damaging effects in peripheral and central nervous systems, kidneys, and blood pressure. If exposures exceed tolerable levels, lead is dangerous to individuals of any age, while fetuses and young children are the most vulnerable members. Based on the Substance Priority List of Agency for Toxic Substances and Disease Registry (ATSDR) 2013 Substance Priority List, Pb(II) is the top 2 hazardous material of environmental concern with high frequency, persistency, and toxicity [4, 5]. The methods have been applied for Pb(II) removal in contaminated water mainly including chemical precipitation, electrochemical reduction, ion-exchange, membrane separation, and sorption [6, 7]. Among these technologies, adsorption is one of the economic and efficient methods for removal of heavy metal from wastewaters. Nanomaterials have extremely small size and high surface area to volume ratio that provided immense scope and opportunities for water decontamination [8, 9].

Sodium alginate is a relatively economical biopolymer with compositions of α-1, 4-l-glucuronic acid (G units) and poly-β-1, 4-d-mannuronic acid (M units) which can provide rich ester groups (–COO^−^) for adsorbing cation [10–12]. Recently, nano alginate materials have attracted significant attentions in the area of heavy metal adsorption [13–15]. When exposed to aqueous calcium ion, sodium alginate will form a three-dimensional gel network, which is essential for adsorption [16].

Quercetin is a naturally-derived polyhydroxy (3, 3', 4', 5, 7-OH) compound, which can form complexes with many metal cations. It is presumed the hydroxyls on quercetin may enhance the adsorption capability of alginate. Besides, we have reported a microcapsule system based on alginate for Cr(VI) removal in our previous research [17]. And the size of the microcapsule is about 200 μm. Considering that small particles (<1 μm) have a larger specific surface area and a higher mechanical strength, it is significant to prepare alginate particles in nanometer. An approach of alginate-in-water (W/O) nano-emulsions coupled with external gelation will be applied to prepare nanoparticles. The present work is aimed at the design of both uniformly sized alginate nanoparticles (AN) and quercetin-decorated alginate nanoparticles (Q-AN) for Pb(II) removal. Batch adsorption experiments were conducted to investigate the removal of Pb(II) from aqueous solution by both AN and Q-AN. The effect of parameters such as solution pH, initial Pb(II) concentration, and co-existing metal ions was studied.

## Methods

### Materials

Sodium alginate (low viscosity, 4–12 cP), Span 80, and Tween 80 were provided by Sigma-Aldrich Co. (St. Louis, USA). Quercetin was supplied by Sangon Biotech, Co., Ltd. (Shanghai, China). Lead nitrate (Pb(NO_3_)_2_), manganous nitrate (Mn(NO_3_)_2_), cobaltous nitrate (Co(NO_3_)_2_), and cadmium(II) nitrate (Cd(NO_3_)_2_) were purchased from Aladdin Industrial Corporation (Shanghai, China). All remaining chemicals used in this study were analytical grade or ACS grade. All solutions were prepared with ultrapure water.

### Preparation of AN and Q-AN

#### Preparation of AN

The AN in aqueous phase of W/O nano-emulsions were prepared by following procedure. (1) 1.05 mL Span 80 and 0.45 mL Tween 80 were added into 150 mL liquid paraffin (HLB = 7.5) in a 500-mL three-necked flask while stirring with a mechanical stirring speed of 500 rpm at a constant temperature of 40 °C for 30 min. (2) To obtain small-sized alginate nanoparticles, the stirring speed was increased to 1000 rpm. 45 mL of 0.50 % (*v*/*w*) alginate was dropped into above liquid paraffin at a rate of 1 mL 6 min^−1^. The solution was then stirred at the same temperature for 60 min. (3) CaCl_2_ (0.10 %) (*v*/*w*) prepared with 60 % (*v*/*v*) ethanol was dropped into the above mixture at a rate of 1 mL 6 min^−1^. Keep stirring for another 60 min. Then, the alginate nano-emulsions was transferred into Eppendorf tubes, centrifuged at 3800 r min^−1^ for 10 min, and then left for equilibration at 25 °C. The upper phase (oil of surfactant or reverse micelles) of the phase-separated samples was discarded, whereas the lower phase containing alginate nanoparticles was collected [18]. (4) An equal volume of ethyl ether was added into alginate nanoparticles and vibrated gently. And the upper phase (solution of ethyl ether and residual liquid paraffin) was removed. Purge lower phase with nitrogen gas to replace ethyl ether. The obtained alginate nanoparticles were filtrated with a 0.22-μm membrane filter and lyophilized or kept in a refrigerator until further investigation.

#### Preparation of Q-AN

The procedure of Q-AN preparation was similar to that of AN except an addition of 0.0675 g quercetin into 45 mL of 0.50 %(*v*/*w*) alginate.

### Characterizations

#### Morphology Characterization

Samples for the transmission electron microscopy (TEM; JEOL JEM-2100F, Japan) observation were dispersed in ultrapure water, and two drops were placed on a carbon-coated copper mesh grid. The experiment was carried out after the evaporation of water.

#### Particle Size Determination and Zeta Potential Measurement

The particle size and zeta potential of the samples were estimated by phase angle light scattering (PALS) using Zetasizer Nano-Series (NanoZS, Malvern, UK), controlled by the Dispersion Technology Software (DTS 5.03, Malvern, UK). Samples were diluted with ultrapure water before measurement. In each case, the experiment was carried out three times.

#### Quantification of Alginate and Quercetin

The quercetin in the Q-AN was assessed by measuring the quercetin released from Q-AN in the methanol solution. The yield of alginate and quercetin was calculated as the percent of alginate and quercetin in obtained nanoparticles, which was compared to the initial amount of alginate and quercetin used in the prepared process, respectively.

#### Thermogravimetric Analysis

Thermogravimetric data (TG-DTG-DSC) for samples were obtained using thermogravimetry and differential scanning calorimetry (TG-DSC; STA449F3, Netzsch, Germany). All tests were conducted with sample mass of 2–5 mg at 10 °C min^−1^ over a range of 50–800 °C. The measurements were carried out in Ar atmosphere

#### FT-IR Analysis

FT-IR spectra of samples were recorded on a FT-IR spectrometer (Tensor 27, Bruker, Germany). The spectra were collected in the range of 4000–400 cm^−1^ with a resolution of 2 cm^−1^ at 16 scans per spectrum.

#### X-ray Diffraction Analysis

The crystal structures of samples were identified using X-ray diffractometer (XRD, D/max-2500, Rigaku, Japan) operated at 50.0 kV and 180.0 mA with Cu Kα radiation, and in the range of 10°–50° with a scanning rate of 0.2° min^−1^.

#### X-ray Photoelectron Spectrometer

The valence state of Pb absorbed on Q-AN was determined by X-ray photoelectron spectrometer (PHI 1600, PerkinElmer, USA). Experiments of Q-AN and adsorption of Pb(II) in this characterization was conducted in nitrogen atmosphere to avoid interference of oxygen in air. Measurement was carried out with an AlK (*hλ* = 1667.0 eV) X-ray source at 250.0 W and pass energy of 29.35 eV for high-resolution analyses. Vacuum in analysis chamber was kept below 3 × 10^−6^ Pa during measurement. Binding energy of spectra was calibrated with aliphatic carbons C1s (284.6 eV).

### Batch Experiments

Batch adsorption experiments for Pb(II) removal in aqueous solution were performed in polypropylene bottles with a reaction volume of 45 mL at room temperature (25 ± 1 °C). Nanoparticles (0.2 g) were sealed in dialysis bags (MW 12,000) and then immersed in the above Pb(II) aqueous solution. Equilibrium adsorption was achieved by a shaker incubator at 120 rpm for 4 h. Effects of several parameters, such as initial pH (2, 3, 4, 5, 6, 7), initial Pb(II) concentration (250, 500, 750, 1000, 1250 mg L^−1^), and co-existing heavy metal ions (Mn, Co, Cd), on removal of Pb(II) in aqueous solution were studied. The solution pH was adjusted with 0.1 M HCl and 0.1 M NaOH. The adsorption capability of AN and Q-AN was conducted in 1000 mg L^−1^ Pb(II) aqueous solution at pH 7. Experiments of Pb(II) desorption were carried out in 1 M HCl solution. And the AN and Q-AN used in desorption experiment reached the equilibrium adsorption in 500 mg L^−1^ Pb(II) aqueous solution at pH 7. The residual Pb(II) aqueous solution was filtrated with 0.22 μm membrane filter, and then, Pb(II) concentration was quantified with an inductively coupled plasma atomic emission spectroscopy (SPECTRO ARCOS EOP, Spectro Analytical Instruments GmbH, USA).

The percentage of Pb(II) adsorption by nanoparticles was calculated as follows:

1 $$ Q\left(\%\right)=\frac{C_0-{C}_i}{C_0}\times 100\% $$

The absorption capacity of nanoparticles was calculated as follows:

2 $$ {q}_t=\frac{V\left({C}_0-{C}_{\mathrm{i}}\right)}{W} $$

where *C*_0_ is the initial concentration of Pb(II) in the aqueous solution (mg L^−1^) and *C*_i_ is the concentration of Pb(II) after equilibrium adsorption (mg L^−1^). *V* is the volume of solution (L), and *W* is the mass of the nanoparticles used (g). All experiments were performed in triplicate to ensure the precision of the results.

## Results and Discussion

### Characterization of AN and Q-AN

#### Morphology

**Fig. 1:**
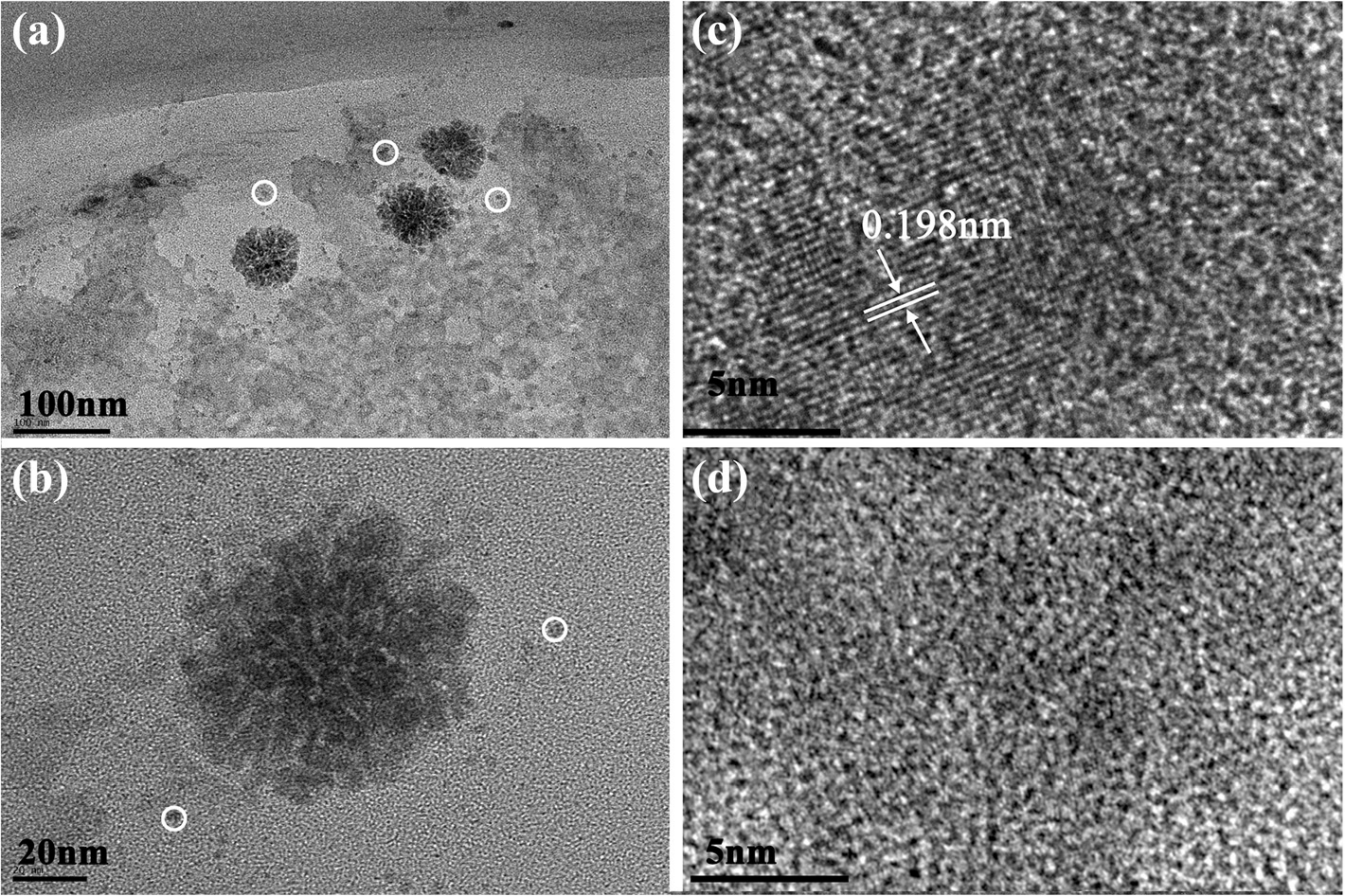
**a** TEM image of Q-AN. **b** A single TEM image of Q-AN. **c** High-resolution TEM image of Q-AN. **d** High-resolution TEM of AN

TEM micrographs of Q-AN were given as examples in Fig. 1. Figure 1a and b indicated that Q-AN was constituted by small primary particles with a diameter of about 1–2 nm (indicated by white circles) [19]. High resolution of TEM in Fig. 1c showed lattice fringes which indicate the presence of microcrystallites. And the various directions of lattice fringes further demonstrated the existence of small primary particles. Meanwhile, no clear crystallinity was observed in the high-resolution TEM image of AN (Fig. 1d). It illustrated the influence of quercetin to the crystal structure of Q-AN. It was noteworthy in Table 1 that the diameter of Q-AN was strikingly smaller than that of AN. And the decline of PDI [20] showed improvement of the uniformity nanoparticle size distribution obviously. It is presumed that the hydroxyl groups in quercetin can form an intermolecular hydrogen bond with alginate and share the coordination site in Ca^2+^ with it as well. The loading rate and encapsulation efficiency of quercetin in lyophilized Q-AN were 0.36 and 10.67 %, respectively.

**Table 1:** Related information about AN and Q-AN

	Diameter (nm)	PDI^a^	Loading rate (%)	Encapsulation efficiency (%)	Adsorption Capability (mg/g)	Desorption rate (%)
AN	95.06 ± 9.97	0.34 ± 0.11	–	–	147.02 ± 3.91	83.26 ± 7.46
Q-AN	58.23 ± 2.34	0.15 ± 0.03	0.36 ± 0.03	10.67 ± 0.89	140.37 ± 5.04	90.07 ± 3.39

^a^PDI is an abbreviation of polydispersity index. PDI < 0.2 corresponded to a homogeneous distribution

#### TG-DTG-DSC

**Fig. 2:**
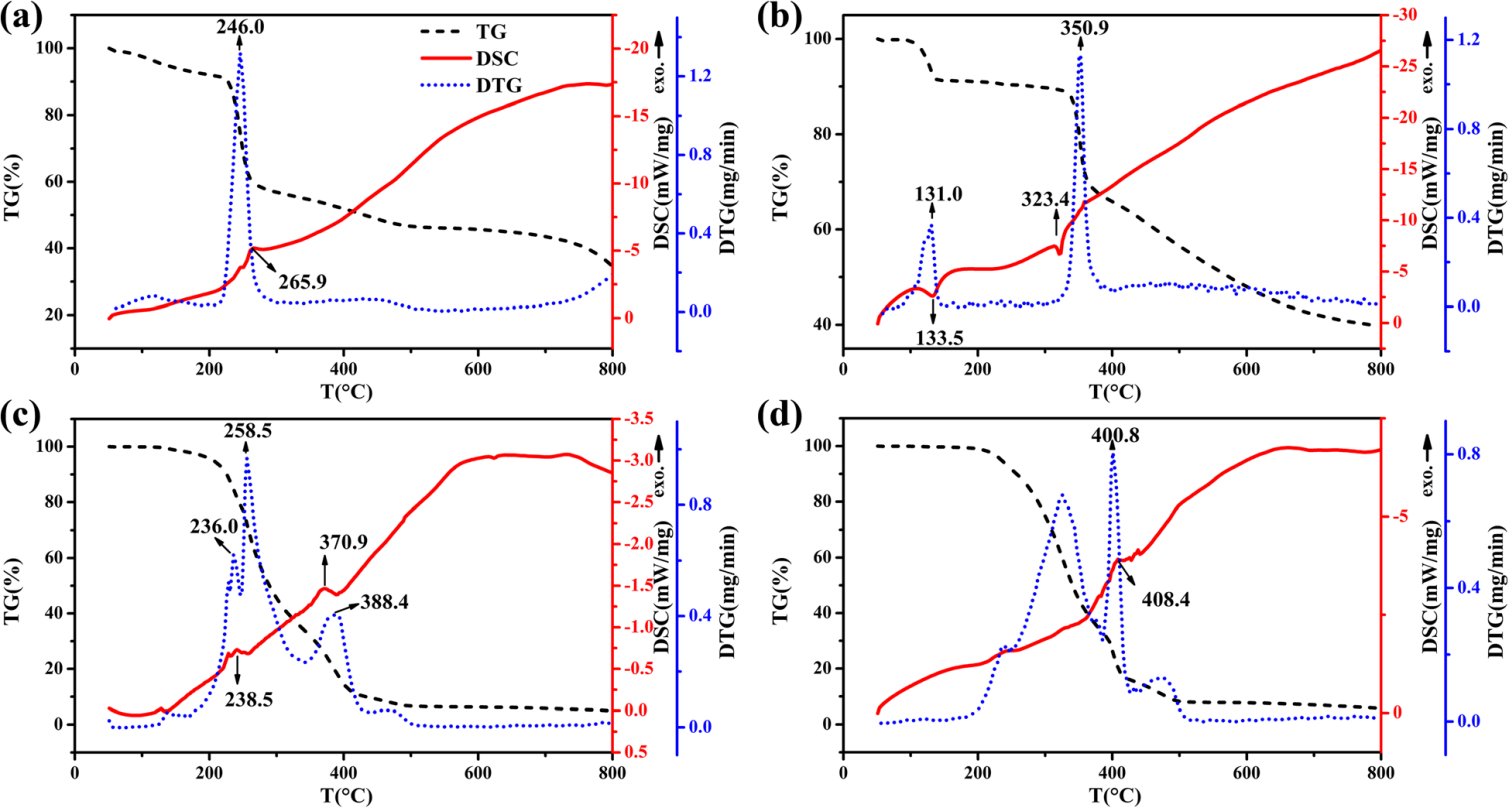
TG-DTG-DSC curves of **a** sodium alginate, **b** quercetin, **c** AN, and **d** Q-AN

The thermogravimetry was used to study the isolated polyelectrolyte alginate and its complex form. TG-DTG curves in Fig. 2a indicated that sodium alginate subjected to a slight weight loss in 50.0–195.9 °C range and then a rapid one in 195.9–576.0 °C range. The first weight loss was mainly caused by the loss of water molecules in the sodium alginate powder, and the second one was due to the degradation of Na-alginate backbone [21]. Two clear weight loss stages of quercetin appeared in 50.0–178.5 °C and 178.5–421.0 °C ranges (Fig. 2b), which were similarly due to its water loss and structure degradation. In TG-DTG curves of AN (Fig. 2c), the slight weight loss in 50.0–138.4 °C range was attributed to the residual moisture in AN after the lyophilization process. However, a sustained and rapid weight loss in 138.5–513.5 °C range illustrated the obvious change of the existing forms of sodium alginate in AN (compared with the TG curve in Fig. 2a). This result was further confirmed by the shift of DTG peak form 246.0 °C (sodium alginate) to 258.5 °C (AN). The TG curve of Q-AN underwent a continued weight loss in 50.0–543.3 °C which demonstrated the thermal decomposition of quercetin [22]. And the intensive DTG peak at 400.8 °C illustrated the improvement of the thermal stability of quercetin by interaction with alginate gel.

The DSC curve of alginate presented one exothermic peak at 265.9 °C corresponding to the thermal decomposition. As to quercetin, the endothermic peak at 133.5 °C was related to the water loss and the second one at 323.4 °C to its melting point. The difference between DSC curve of sodium alginate and AN was mainly caused by gel formation. Two exothermic peaks at 238.5 and 370.9 °C were respectively attributed to the decomposition of gel and residual substances mentioned in DTG analysis. However, no obvious melting point appeared in the DSC curve of Q-AN though the typical crystal structure of quercetin. Besides, the very weak exothermic peak at 408.4 °C might be due to the decomposition of quercetin, and this decomposition temperature is higher than it in the DSC curve of pure quercetin. Thus, it confirmed the interactive force between alginate gel and quercetin.

#### FT-IR

**Fig. 3:**
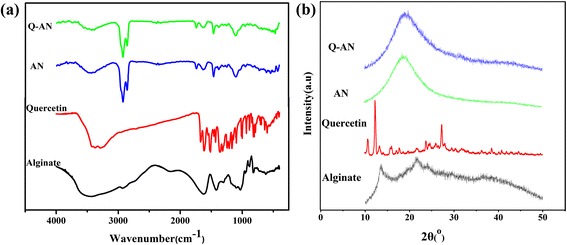
**a** FT-IR spectra and **b** X-ray diffraction spectra of sodium alginate, quercetin, AN, and Q-AN

One approach to provide information on molecular interaction of nanoparticles was to monitor changes in the FT-IR spectra of their component parts. The FT-IR spectra of sodium alginate, quercetin, AN, and Q-AN are shown in Fig. 3. In the FT-IR spectra of alginate (Fig. 3a), the major peaks observed were as follows: a strong-broad band at 3435.09 cm^−1^ (O–H stretching), a weak peak at 2925.81 cm^−1^ (C–H stretching), a medium-sharp peak at 1623.60 cm^−1^ and a medium-shoulder peak 1419.73 cm^−1^ (asymmetric and symmetric COO–stretching), and then peaks at 1109.22 cm^−1^ and 1029.25 cm^−1^ (C–O stretching [6]).

For quercetin (Fig. 3b), the major peaks were as follows: a strong-broad band at 3422.82 cm^−1^ (O–H stretching), a medium-sharp peak at 1668.93 cm^−1^ and a strong-sharp 1610.88 cm^−1^ (C = O stretching), a strong-sharp peak at 1523.81 cm^−1^ (aromatic group), a medium-sharp peak at 1316.49 cm^−1^ and a medium-sharp peak at 1163.08 cm^−1^ (C–O–C stretching), and a weak-sharp peak at 930.89 cm^−1^ (aromatic group C–H stretching).

When the alginate nanogel was formed in the presence of Ca^2+^, several shifts of absorbance peaks were observed compared with the FT-IR spectra of alginate. The change was observed for C–O–C stretching peak at 1109.22 cm^−1^ (sodium alginate) to 1101.10 cm^−1^ (AN). A very slight shift of COO– asymmetric stretch peak at 1623.60 cm^−1^ (sodium alginate) to 1627.58 cm^−1^ (AN) was also observed. However, the COO– symmetric stretch peak at 1419.73 cm^−1^ (sodium alginate) was distinctly transferred to 1463.04 cm^−1^ (AN). Peak shifts mentioned above provided the evidence that the Na^+^ ions in the sodium alginate were replaced by Ca^2+^, thus forming a regular three-dimensional net structure bound by Ca^2+^ [16]. The FT-IR spectra of Q-AN (Fig. 3d) indicated that it kept the major absorption peaks of AN, namely C–O–C stretching peak and COO– asymmetric and symmetric stretch peaks. However, peaks of quercetin were concealed. The reason might be that the trace of quercetin was involved in the interaction with alginate molecule in the preparation process.

#### XRD

X-ray diffraction was performed to study the crystallinity of samples. The characteristic peaks of sodium alginate (Fig. 3b) rose at 13.56 °, 20.74 °, 21.64 °, 24.06 °, 28.96 °, 36.64 °, and 38.40 ° (2θ), demonstrating its semi-crystalline nature [23]. The crystalline nature of quercetin was confirmed by distinct peaks at 10.70 °, 12.32 °, 13.36 °, 16.02 °, 17.76 °, 23.76 °, 27.36 °, 38.6 °, and 40.84 ° [24, 25]. However, the AN and Q-AN exhibited no distinct peaks but broad ones. Similar phenomena of microcrystallites observation in amorphous structure were also reported by some research [26–28]. One of the reasons for this is that the gelation processes changed the spatial structure of sodium alginate. And another reason accounted for the intermolecular interaction occurring between quercetin and sodium alginate. Similar phenomena that oridonin and quercetin transformed from crystal form to amorphous form when loaded in nanoparticles have also been observed by *al* [29, 30]. And this is accordance with the results obtained in TG-DTG-DSC and FT-IR analysis.

### Batch adsorption of Pb(II)

#### Effect of pH

**Fig. 4:**
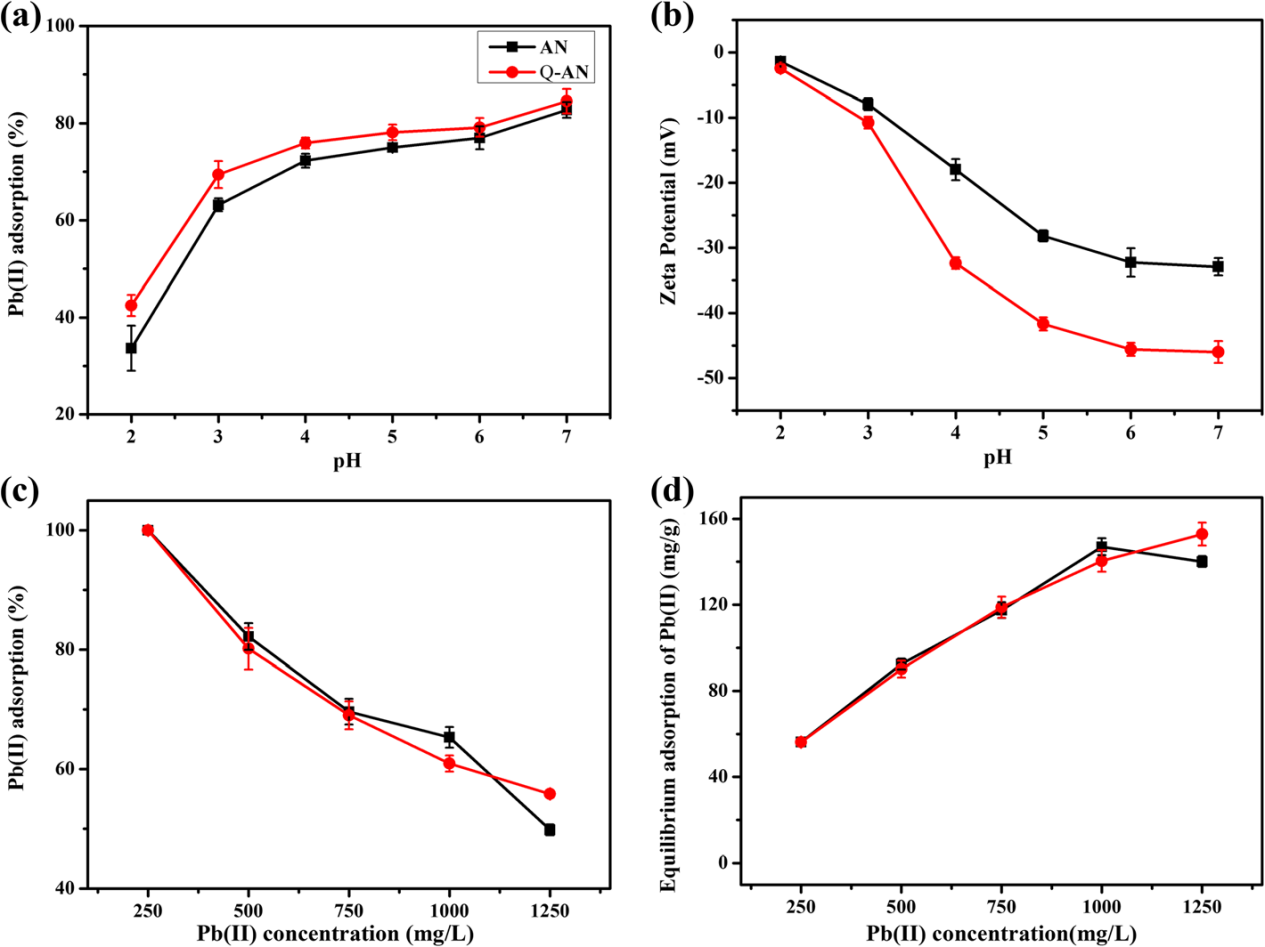
**a** The effect of pH on the adsorption of Pb(II) ions; the initial concentration of Pb(II) ions was 500 mg L^−1^. **b** The effect of pH on the Zeta potential of AN and Q-AN; the initial concentration of Pb(II) ions was 500 mg L^−1^. **c** The effect of initial Pb(II) concentration on the adsorption of Pb(II); the initial pH was 7. **d** The effect of initial Pb(II) concentration on the equilibrium adsorption of Pb(II); the initial pH was 7

As shown in Fig. 4a, the Pb(II) adsorption rate increased from 33.66 to 82.74 % for AN and 42.46 to 84.52 % for Q-AN with the increase of pH from 2 to 7 (Eq. (1)). It was known that Pb^2+^, Pb(OH)^+^, Pb(OH)_2_^0^, and Pb(OH)_3_^−^ were frequently found in aqueous solution [31]. In acidic medium, Pb^2+^ was the major lead species. And a small portion of Pb(OH)^+^ was present with the pH increased to neutral condition [32]. At low pH, a high H^+^ concentration competed with Pb(II) cations for active sites on the surface of AN and Q-AN. Besides, the surface of AN and Q-AN turned into a negative charge when pH increased [33–35]. And it was confirmed by estimating the Zeta potential of AN and Q-AN at pH 2–7. Figure 4b indicates an obvious decrease of Zeta potential from −1.41 to −32.94 mV (AN) and −1.99 to −44.70 mV (Q-AN) when pH enhanced from 2 to 7. Namely, the more negative the nanoparticles are charged, the stronger is the electrostatic attraction between them and Pb(II). Therefore, an increasing acidic medium facilitated Pb(II) adsorption by affecting both the lead species and adsorbent surface.

#### Effect of Initial Pb(II) Concentration

**Fig. 5:**
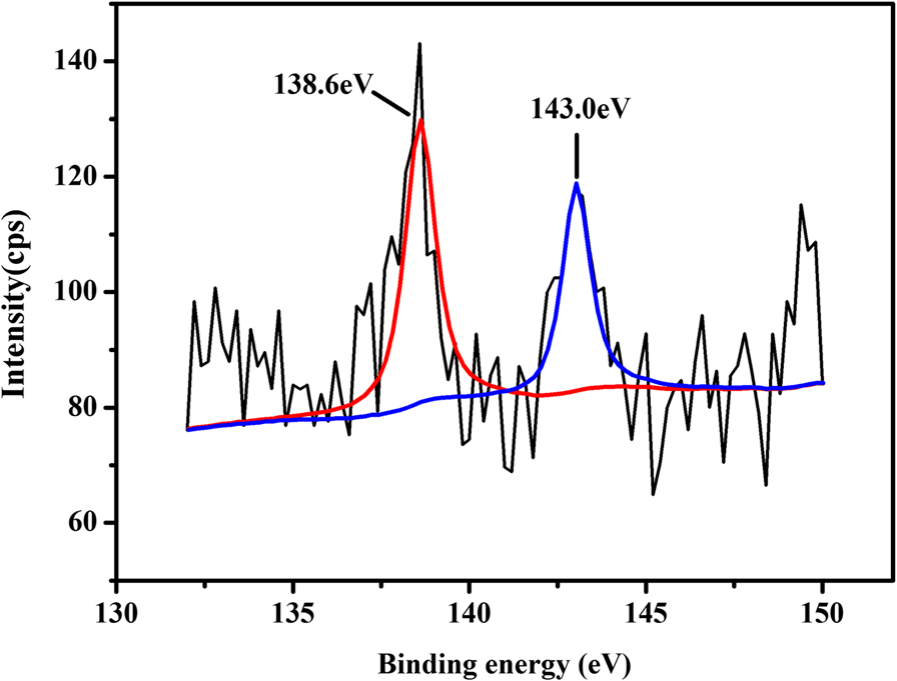
XPS spectra of Pb(II)-adsorbed Q-AN

Adsorptive removal of Pb(II) at various initial concentration from 250 to 1250 mg L^−1^ was also studied. As illustrated by AN and Q-AN adsorption curves (Fig. 4c), the Pb(II) adsorption rate decreased with the increase of initial Pb(II) concentration. It was attributed to the enhanced quantity of Pb(II) competition for the limited adsorption sites, and thus, the adsorption rate decreased at a higher initial Pb(II) concentration. However, Fig. 4d demonstrated that a higher initial Pb(II) concentration gave rise to a higher equilibrium adsorption capacity. The reason was that superfluous Pb(II) made sure of a more efficient use of the adsorbent. The mechanism for Pb(II) adsorption was further studied by investigating the XPS spectra of Pb(II)-adsorbed Q-AN (Fig. 5). The high-resolution spectra showed two peaks at 138.6 and 143.0 eV, which were primarily attributable to binding energies for Pb 4f7/2 and Pb 4f5/2 orbital, respectively [36]. Thus, it demonstrated the existence of Pb^2+^ (Pb–O bond), and this was consistent with previous reports [11, 37].

#### Effect of Other Co-existing Metal

**Fig. 6:**
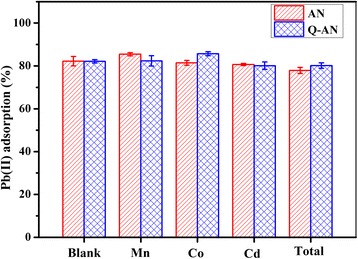
The effect of co-existing metal on the adsorption of Pb(II). The initial concentrations of Pb(II) and co-existing metal were 500 and 100 mg L^−1^, respectively. The initial pH was 7

**Fig. 7:**
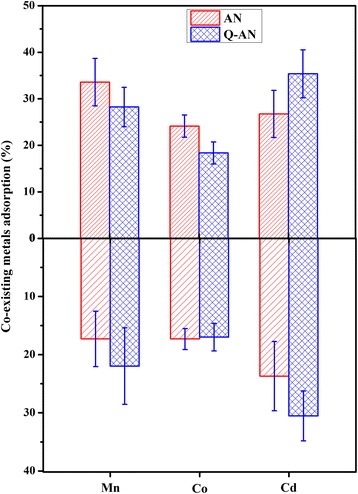
The adsorption rate of co-existing metal. The upper part was only the single co-existing metal present in the solution. The bottom half was all three co-existing metals present in the solution. The initial concentrations of Pb(II) and co-existing metal were 500 and 100 mg L^−1^, respectively. The initial pH was 7

Co-existing metal might compete with Pb(II) for the adsorption sites on the adsorbents; thus, three typical heavy metals, Mn, Co, and Cd, were added into the reaction solution to estimate their effects on Pb(II) adsorption. As shown in Fig. 6, the presence of examined metals with a concentration of 100 mg L^−1^ only caused a 10–20 mg L^−1^ decrease in removal rate of Pb(II). In Fig. 7, about 20–40 mg L^−1^ decrease of the co-existing metals was observed. In the presence of a single metal, a 80.10–85.66 % absorption rate was achieved by both AN and Q-AN. However, when Mn(II), Co(II), and Cd(II) were co-existing, the AN group got an absorption rate of 77.86 %, which was 2.29 % lower than that in the Q-AN group. Generally, the results illustrated that both the AN and Q-AN were effective adsorbents for Pb(II) in aqueous solution, and the presence of abovementioned ions had slight impact on Pb(II) adsorption [11, 23].

### Adsorption Capability and Desorption Rate

The adsorption capability of AN and Q-AN for Pb(II) were 147.02 and 140.37 mg L^−1^ (Eq. (2)). Comparatively, the maximum adsorption capacity of sodium alginate-related nano-adsorbents has been listed in Table 2. The data reported in Table 2 illustrated that AN and Q-AN was comparable to or higher than some of the other adsorbents. The desorption experiment was also conducted to estimate the recovery rate of Pb(II) absorbed on AN and Q-AN. Before the desorption experiment, the AN and Q-AN used for this desorption experiment firstly reached the equilibrium adsorption in 500 mg L^−1^ at pH 7. And the desorption experiment was carried out in 0.1 M HCl [38, 39]. The amount of Pb(II) desorbed was calculated, and the desorption rates of AN and Q-AN were 83.26 ± 7.46 % and 90.07 ± 3.39 % (Table 1), respectively.

**Table 2:** Maximum adsorption capacity of sodium alginate related nano-adsorbents for Pb(II) removal from aqueous media

Sodium alginate-related nano-adsorbents	Maximum adsorption capacity (mg/g)	Reference
Silica nanopowders/alginate composite	83.33	[15]
TSTC [4] AS-s-SA nanogel	16.9	[23]
Superparamagnetic Fe_3_O_4_@TSTC [4] AS-s-SA nanocomposite	19.96	[23]
Magnetic alginate beads based on maghemite nanoparticles	50	[40]
Polyaniline nanofibers assembled on alginate microsphere	251.25	[41]

## Conclusions

In this study, AN and Q-AN were prepared with the technique of nano-emulsions coupled with external gelation. These nanoparticles turned out to be effective for the removal of Pb(II) from aqueous solutions. Compared with the diameter of AN, that of Q-AN underwent an obvious decrease from 95.06 to 58.23 nm. The removal of Pb(II) is found to be highly pH-dependent, and the optimal pH value was 7 among examined pHs. Under the conditions of 1000 mg L^−1^ Pb(II) at pH 7, the adsorption capability of AN and Q-AN were similar, which were 147.02 ± 3.91 mg g^−1^ and 140.37 ± 5.04 mg g^−1^, respectively. The Pb(II) desorption rate of AN and Q-AN were 83.26 ± 7.46 % and 90.07 ± 3.39 %, respectively. Generally, Q-AN got a slightly higher quality for Pb(II) removal than AN. Though further research is required to illustrate the mechanism of marked nanoparticle diameter decrease after the decoration of quercetin, the satisfactory Pb(II) removal rate illustrated that AN and Q-AN are promising adsorbents, which are probably to obtain wide application.
